# Integration of Disability Awareness Improves Medical Students’ Attitudes Toward People with Disabilities

**DOI:** 10.1007/s40670-024-02004-0

**Published:** 2024-03-09

**Authors:** Tanvee Sinha, Ashley Parish, Donald H. Lein, Elizabeth Wylie, Cathy Carver, William S. Brooks

**Affiliations:** 1 Marnix E. Heersink School of Medicine, University of Alabama at Birmingham, Birmingham, AL USA; 2 Department of Physical Therapy, School of Health Professions, University of Alabama at Birmingham, Birmingham, AL USA; 3 Spain Rehabilitation Center, University of Alabama at Birmingham, Birmingham, AL USA; 4 Department of Cell, Developmental & Integrative Biology, Marnix E. Heersink School of Medicine, University of Alabama at Birmingham, 1720 2nd Ave. S, Volker Hall 611, Birmingham, AL 35294-0019 USA

**Keywords:** Disability, Disability awareness, Health disparities, Interprofessional education, Wheelchair

## Abstract

Persons with disabilities (PWD) often require special accommodations and more comprehensive healthcare yet frequently have encounters with physicians who are unprepared to provide tailored and complete care. A multipronged disability awareness curriculum for second-year medical students was implemented, including content on disability etiquette, patient-centered and interprofessional learning sessions for individuals with physical disabilities and intellectual and developmental disabilities, and a debriefing session with physiatrists. The objective of this study was to utilize a mixed methods approach to evaluate the disability awareness curriculum in undergraduate medical education (UME). Assessment was conducted using course evaluations, pre- and post-surveys including the Attitudes and Perspectives Towards Persons with Disabilities (APPD) scale and Multidimensional Attitudes Scale Toward Persons with Disabilities (MAS), and student focus groups. The mean scores from both the APPD (2.11 ± 0.43 pre-score vs. 1.7 ± 0.39 post-score) and MAS (2.45 ± 0.43 pre-score vs. 2.25 ± 0.55 post-score) indicate the curriculum improved medical students’ attitudes toward PWD (*p* < 0.05), with lower numbers representing more favorable attitudes. After completing the curriculum, medical students’ attitudes were comparable to those of doctor of physical therapy (DPT) students. Qualitative analysis from focus groups highlighted four major themes: education, comfort level, impact on future practice, and disability differences. This curriculum has potential as a valuable framework for delivering effective disability education to medical students to prepare future physicians to serve PWD and their unique needs. It meets core competencies, provides an opportunity to learn in interprofessional environments, and integrates PWD into the educational process.

**Supplementary Information:**

The online version contains supplementary material available at 10.1007/s40670-024-02004-0.

## Introduction

Over one quarter of adults in the USA have a disability that impacts overall health, making persons with disabilities (PWD) the largest minority group who requires skilled medical services [1, 2]. Due to their challenges and complex medical needs, PWD require unique accommodations in their healthcare, yet they are more likely to have a clinical encounter where their healthcare provider does not carefully listen to them, show respect, or perform a comprehensive physical examination [3]. One study found that only 40.7% of physicians were comfortable providing quality care to PWD, and just 56.6% were welcoming to those with a disability [4]. Physicians perceive the lack of accessible facilities and equipment to be barriers to their ability to provide care for PWD in general and those with mobility impairments, specifically [5, 6]. Other barriers that have been noted include limited resources for communication, limited knowledge and experience, challenges within the healthcare system, and provider bias [4, 7]. Many PWD develop conditions that could be avoided if managed with effective care [8]. Furthermore, disability professionals, those who have dedicated work with PWD or about disability, have both explicit and implicit preferences for nondisabled people [9]. These findings of bias and discomfort with PWD emphasize the need to ensure adequate disability awareness education across the medical education continuum.

Undergraduate medical education (UME) offers an early opportunity to address the skills and attitudes required of physicians to effectively care for this population. However, the proportion of American medical schools that include a disability awareness curriculum may be as low as 23%, which results in a sense of unpreparedness among medical students in caring for PWD [10, 11]. Further complicating this lack of preparation and training, conflicting views on how to define disability awareness programs exist [10]. The Association of American Medical Colleges (AAMC) allows medical schools academic freedom in determining how to incorporate cultural competency, healthcare disparities, and treatment of PWD into curricula [12]. For example, some schools utilize standardized patients to simulate physical examination skills and communication with a PWD, while others have incorporated structured clinical examinations during clerkship years with PWD to give students hands-on experience with this population [3, 13]. Currently, there is no appreciable disability curriculum that is followed by all medical schools and/or fully encompassing of how to approach care for PWD [14].

Further complicating the challenges of providing disability education is the fact that disability comes in a variety of forms. The Centers for Disease Control and Prevention classify disability into six categories, (1) mobility, (2) cognition, (3) independent living, (4) hearing, (5) vision, and (6) self-care, with impairments in mobility and cognition each affecting approximately 12% of US adults [2]. Due to the unique needs of PWD within each category, comprehensive disability awareness curricula are challenging to implement within already crowded medical school curricula.

Recently, we reported on Come Roll with Me (CRWM), a curriculum designed to increase awareness of and empathy toward PWD, specifically wheelchair users [15]. Qualitative evaluation of this experience showed that medical students displayed increased awareness of barriers that wheelchair users experience when accessing healthcare and greater empathy toward wheelchair users. Doctor of physical therapy (DPT) students, who served as interprofessional peer educators and instructed medical students about wheelchair mobility and transfers, also reported improved attitudes toward wheelchair users even after having completed two-thirds of a curriculum replete with content related to disability as mandated by the profession’s accreditation body [16]. The success of CRWM may be attributable to the use of previously proven education strategies such as the use of wheelchair users as facilitators [17, 18] and interprofessional peer-assisted learning [19–21]. CRWM focused on physical disability, which represents the greatest fraction of PWD but is not inclusive of all disability types [2]. The purpose of this study was to expand upon CRWM to develop, implement, and evaluate through a mixed methods approach a multicomponent disability awareness curriculum within a UME preclinical course. We hypothesized medical students’ attitudes toward PWD would improve after completing this disability awareness curriculum.

## Materials and Methods

### Disability Awareness Curriculum

The Musculoskeletal and Skin Module is a 6-week course in the second year of the preclinical phase of the organ system-based curriculum at the Marnix E. Heersink School of Medicine [22]. The course provides instruction on the normal and abnormal functions of the integumentary, skeletal, and muscular systems as well as a survey of the most common rheumatologic diseases and pharmacotherapy for these conditions. Curricular elements embedded within the module guide students in meeting the course learning objective “Develop empathy for patients with disabilities” (Table 1). Some elements including “Come Roll with Me” were piloted in 2021 [15] and then expanded upon for the 2022 course reported here.

**Table 1:** Disability curricular elements

**Activity**	**Description**	**Length**
Didactic lecture	Communication etiquette with PWD	1 h
Come Roll with Me	Interprofessional experience with DPT students and wheelchair users from the community to learn about health disparities for PWD	2 h
United Ability	One of three activities at a local non-profit agency that provides day services for IDD: 1) Destigmatizing going to the doctor 2) Prepare a healthy meal 3) Exercise	2 h
Debrief	Small group debrief led by faculty in the Department of Physical Medicine and Rehabilitation	1.5 h
Patient presentations	Two large group patient presentations associated with content on muscle disease and skeletal dysplasia	2 h

*PWD* persons with disabilities, *DPT* doctor of physical therapy, *IDD* intellectual and developmental disability

First, students were provided a didactic lecture on communication etiquette when interacting with PWD. The objectives for this lecture included: (1) Discuss unique considerations for PWD, (2) consider the impact of implicit biases toward PWD, (3) discuss patient-centered communication strategies, and (4) develop empathy for patients with disabilities. Second, students participated in CRWM, a 2-h interprofessional experience that utilizes DPT students and wheelchair users from the community to teach medical students about communication with and health disparities experienced by individuals with physical disabilities [15]. Third, students spent 2 h at a local non-profit organization, United Ability, which provides day activities for individuals with intellectual and developmental disabilities (IDD). While at United Ability, students engaged in one of three activities: (1) destigmatizing going to the doctor, (2) preparing a healthy meal, or (3) exercise. Students performed (1) by discussing common scenarios that would require going to the doctor and role playing these conversations or listening to participant concerns about seeking medical care; students attempted to establish trust and build rapport with participants in a safe space. For preparing a healthy meal, students assisted participants with preparing oatmeal using a variety of ingredients and toppings. For the exercise activity, participants engaged in bowling, basketball, and a dance party. Each activity provided opportunities for communication, promoting healthy living, and/or developing fine motor skills.

Following completion of CRWM and the United Ability experience, students participated in a small group debrief led by faculty in the Department of Physical Medicine and Rehabilitation. Finally, students attended two 1-h, large-group patient presentations associated with content on muscle disease and skeletal dysplasia. For the myopathy patient presentation, individuals from the community with differing muscle diseases presented their experiences and diagnoses. For the skeletal dysplasia patient presentation, a pediatric patient with achondroplasia and her guardian discussed their experiences with healthcare and clinical trials for the drug vosoritide [23, 24]. Each patient presentation concluded with a session debrief for students to reflect with one another on the encounter.

### Recruitment and Survey Distribution

Wheelchair users were recruited to participate in CRWM by one author (C.C.) who works as a physical therapist at a local Wheelchair and Seating Clinic. Participants were invited by email to assist with CRWM as a facilitator and were compensated with $100 USD for each day of participation. Funding was provided by the Office of Undergraduate Medical Education. Wheelchair users were former patients of C.C. or employees of the university, and each has a long-standing partnership with the CRWM community program.

While enrolled in the 2022 Musculoskeletal and Skin Module, second-year medical students were invited to participate in a research study to evaluate the impact of a disability awareness curriculum embedded within the course. A verbal invitation was made during the course introduction by the course director, and two follow-up invitations were distributed via email to the class. Second-year DPT students participated in CRWM as interprofessional peer educators. DPT students were invited to participate in the research study through verbal invitation by a study author followed by two invitations distributed via email to the class. Students who consented to participation and completed both the pre- and post-survey were provided a research incentive in the amount of $10.00 USD.

Participating students were provided a link to a Qualtrics (Provo, UT) survey, which included one item asking students to consent to their participation in the research study, the 14-item Attitudes and Perspectives Towards Persons with Disabilities (APPD) Scale [25], and the 34-item Multidimensional Attitudes Toward Persons with Disabilities Scale (MAS) [26]. The newly developed APPD, which measures individuals’ social attitudes toward PWD using four subdomains, community integration, discomfort, charitability, and sense of burdening, is reported to have good internal consistency (Cronbach’s α = 0.84 with scales ranging from 0.64 to 0.85), content validity, and construct validity [25]. The MAS, which measures attitudes toward PWD in three domains, affect, cognition, and behavior, also has good internal consistency (Cronbach’s α of all domains ranged from 0.83 to 0.90), content validity, and construct validity as well as concurrent validity [26]. This scale aims to assess situational attitudes upon meeting a person with a physical disability for the first time. The pre-survey link was distributed to medical students during the first week of the Musculoskeletal and Skin course and to DPT students two weeks prior to CRWM. The post-survey link was distributed to medical students after completion of the 6-week course and to DPT students immediately following CRWM. The study was granted approval by the UAB Institutional Review Board (Protocol #300007921).

### Focus Groups

Among the medical students who participated in the disability awareness curriculum, twelve individuals were agreeable to attend one of two semi-structured focus groups to better understand which elements of the curriculum were most impactful and why. These focus group participants were provided a research incentive in the amount of $20.00 USD. Both the survey and focus group incentives were provided from departmental funds available to the corresponding author.

Focus group sessions were held on Zoom (San Jose, CA), limited to 1 h in length, and led by one investigator experienced in leading focus groups (D.H.L.). Questions were derived to explore experiences, perceptions, and reflection of integrated activities. The focus groups were recorded and then transcribed verbatim (Research Transcriptions, Lakewood Ranch, FL). One hundred eighteen minutes of content was used for analysis.

### Data Analysis

Quantitative data obtained from student survey responses were exported from Qualtrics and analyzed with Microsoft Excel. Student’s *T*-test was used to compare mean Likert scale responses for medical and DPT students before and after the study intervention. Significance was set at *p* < 0.05. Cronbach’s α was used to assess the internal reliability of each scale.

A constructivist grounded theory approach was utilized to carry out a thematic analysis of the focus group transcripts. This approach is often used in education to create a new theory regarding a phenomenon using inductive reasoning from qualitative data. It differs from classical grounded theory approaches as the researchers are not neutral observers. With constructivist grounded theory, there is an underlying assumption that involvement of the researchers seeking data from participants is what creates the data and enriches the meaning of data in the context of the participants’ voices [27].

Two authors (T.S. and A.P.) independently read and coded the transcripts using NVIVO (QRS International, Burlington, MA). Open coding was used to identify themes and subthemes by each researcher. A third author (W.S.B) reviewed codes and facilitated discussion to resolve discrepancies prior to generation of a final codebook [28]. The authors set a coding agreement threshold of at least 90% to achieve credibility of data. T.S. and A.P. then independently re-coded the transcript using the final codebook. Intercoder agreement was calculated with NVIVO. The mean Kappa coefficient was 0.37, and percent agreement was 96.8%.

## Results

### Quantitative Evaluation of Medical Students’ Attitudes

Of 172 medical students enrolled in the 2022 Musculoskeletal and Skin Module who participated in the disability awareness curriculum, 61.6% (*N* = 106) and 59.3% (*N* = 102) completed the pre- and post-surveys, respectively. The DPT cohort included 52 students; 82.7% (*N* = 43) and 75% (*N* = 39) completed the pre- and post-surveys, respectively. Response rates exceeded recent recommendations for medical education research [29].

The APPD Scale was administered to medical and DPT students with Cronbach’s α = 0.79. Prior to their engagement with the disability awareness curriculum, medical students demonstrated significantly lower baseline attitudes toward PWD relative to their DPT peers (Table 2). Medical students’ attitudes in all four subdomains of the APPD significantly improved and were equivalent to those of the DPT students after participation in the curriculum. No changes were observed in DPT student attitudes, likely due to their prior curricular coursework related to disability awareness, especially regarding environmental barriers and facilitators.

**Table 2:** Attitudes and perspectives towards persons with disabilities (APPD) scale

	**DPT students**			**MD students**			**MD vs. DPT (*p*-value)**	
	**Pre**	**Post**	***p*-value**	**Pre**	**Post**	***p*-value**	**Pre**	**Post**
**Community integration**	1.34 (0.40)	1.30 (0.40)	0.63	1.49 (0.48)	1.24 (0.31)	< 0.001	0.08	0.34
**Discomfort**	1.46 (0.53)	1.58 (0.46)	0.29	2.1 (0.67)	1.60 (0.6)	< 0.001	< 0.001	0.86
**Charitability**	2.70 (0.86)	2.64 (0.66)	0.74	3.17 (0.75	2.56 (0.61)	< 0.001	0.001	0.58
**Sense of burdening**	1.78 (0.62)	1.79 (0.61)	0.91	2.06 (0.69)	1.79 (0.69)	0.001	0.02	0.96
**Overall**	1.73 (0.40)	1.74 (0.32)	0.91	2.11 (0.43)	1.7 (0.39)	< 0.001	< 0.001	0.62

*DPT* doctor of physical therapy, *MD* doctor of medicine

Internal reliability of the MAS as measured by Cronbach’s α was 0.88. Participation in a disability awareness curriculum closed the observed baseline gap in attitudes between medical and DPT students in a manner similar to that observed by the APPD Scale (Table 3). Medical students demonstrated improved attitudes in two of the three domains of the MAS: affect and cognition.

**Table 3:** Multidimensional attitudes scale toward persons with disabilities (MAS)

	**DPT students**			**MD students**			**MD vs. DPT (*p*-value)**	
	**Pre**	**Post**	***p*-value**	**Pre**	**Post**	***p*-value**	**Pre**	**Post**
**Affect**	2.67 (0.77)	2.55 (0.65)	0.47	2.66 (0.56)	2.42 (0.68)	0.006	0.94	0.30
**Cognition**	1.90 (0.50)	2.02 (0.42)	0.24	2.08 (0.52)	1.91 (0.56)	0.02	0.05	0.26
**Behaviors**	2.28 (0.69)	2.42 (0.53)	0.30	2.48 (0.70)	2.33 (0.72)	0.11	0.11	0.46
**Overall**	2.35 (0.54)	2.36 (0.44)	0.89	2.45 (0.43)	2.25 (0.55)	0.004	0.24	0.23

*DPT* doctor of physical therapy; *MD* doctor of medicine

### Qualitative Assessment of Disability Awareness Curriculum

A subset of medical students participated in one of two semi-structured focus groups to evaluate the disability awareness curriculum and its impact on students. Qualitative analysis yielded four major themes: education, comfort level, impact on future practice, and disability differences. Table 4 provides the themes and subthemes that emerged from the analysis and number of references that were coded to each subtheme. All names provided in the following narrative are pseudonyms to protect the identity of study participants.

**Table 4:** Themes and subthemes of qualitative analysis

**Theme**	**Subthemes**	**No. references**
Education	Improving current curriculum	23
	Continued education of self over time/training	7
	Improving disability education in other settings/expand this to other learners/programs	11
	Benefit of small group interactions	13
	Interprofessional education (IPE)	18
Comfort level	Negative, unprepared for setting	11
	Positive, grew from experience	38
Impact on future practice	Advocate for treating as humans in society	17
	Inform on how they will practice in future	14
	Every patient is unique and requires individualized care	25
Disability differences	Ease of communication depending on disability	15
	Difference in comfort level	12

#### Theme 1: Education

Within the education theme, student comments fell into five subthemes. First, students highlighted the benefits of small group interactions with PWD and the opportunities to have real conversations with them:

*The most impactful part was just being paired with the person. I think being one on one gave me more exposure than being in a group because it really put me on the spot to interact with whoever I was paired with. And, I got to know that person better and also some of the struggles that they may have. (Jason)*

Students also spoke of their desire to *continue to learn about disability and increase access to care for those patients (Josiah)* over the course of their training and professional service. Students also spoke about the need to improve disability education for other learner groups and settings:

*I think [Come Roll with Me] should be something that happens in more settings than just medical students. (Rashad)*

The benefits of interprofessional education (IPE) and working with DPT students were also noted by medical students as an important educational outcome:

*The PT students and faculty were really great and really knowledgeable. It did make me realize how much of a knowledge gap we have in terms of physical therapy in general and a lot of other fields too. And how much we are going to need to learn from and rely on other healthcare professions. (Aaliyah)*

In considering the disability awareness elements of the course, medical student provided many suggestions for improvements in the curriculum:

*Nowhere in either of those modules was cerebral palsy brought up, as well as a myriad of other very common congenital MSK disabilities. So, I’d really like to see that in the curriculum. (Sami)*

#### Theme 2: Comfort Level

The second major theme to emerge from focus groups was around the level of comfort students felt with their educational settings and activities. Students noted a mixture of both positive and negative experiences. Positive experiences were associated with a sense of growth from the experience:

*I feel like some of that anxiety I had before going into it, I don’t feel as anxious anymore just because I learned ways of communicating and making people feel more comfortable. (Rylee)*

Negative experiences were associated with students feeling unprepared for the setting or interactions:

*There were some parts of it where I just felt very uncomfortable because I received no response and sometimes I felt like I was being annoying or I was talking to my partner in a way that he didn’t enjoy me approaching him. I felt like I was not necessarily making his day better. (Leah)*

#### Theme 3: Impact on Future Practice

How the curriculum will impact future clinical practice was the third major theme identified by qualitative analysis. Students spoke about the need for *each person to be cared for individually in a different manner to best optimize their care (Sami)* and the importance of including patients, regardless of their disability, in their own care:

*I think my takeaway was that even for patients with disabilities, and regardless of how severe they are, there’s still an important need for providers to include patients in their care and meet patients where they are. (Divya)*

Advocacy for this patient population in healthcare and in society was another subtheme:

*We are to a large extent our patients’ biggest advocates in the healthcare system. And I think that it’s important for us to have an active role in being cognizant of things that make big impacts on this population so that we can better advocate for them in our facilities and the way that care is delivered. (Aaliyah)*

#### Theme 4: Disability Differences

The fourth theme identified in the analysis was around the differences between individuals with physical disabilities and those with intellectual and developmental disabilities. Most notably, students discussed the challenges in communicating with some individuals with IDD:

*My partner at United Ability used a communication device…I have no idea what her diagnosis was, but to get to interact with her using the communication device was really cool for me and then I spent time after looking into different types of communication devices that allow nonverbal patients to communicate. We were able to have conversations, and she was able to answer my questions. I think I was able to engage her as a human being a lot more. (Jacob)*

## Discussion

The purpose of this study was to evaluate a newly developed, multicomponent disability awareness curriculum within undergraduate medical education to address the unpreparedness of medical students to treat PWD [30]. This novel curriculum was created by expanding the experiential CRWM learning activity, which was previously implemented and qualitatively found to increase awareness of PWD, to a broader curriculum that encompassed a greater breadth of disability types [15]. After participating in the expanded curriculum, medical students reported significantly improved attitudes toward PWD. In addition, qualitative analysis of medical student focus groups supported an increased awareness as well as the desire to learn more about how to treat and advocate for PWD. Qualitative analysis also provided suggestions for improving this new disability awareness curriculum. Based upon these results, the investigators plan to create more one-on-one experiences with PWD during CRWM and provide more didactic content related to the conditions represented by the participants at CRWM and United Ability. Additional resources around disability etiquette and communication will also be provided to students.

Triangulation of data indicates that the medical students’ improved attitudes toward PWD are robust. The APPD [25] and MAS [26] scales have good psychometric properties, and both demonstrated that medical students’ overall attitudes toward PWD significantly improved from baseline to immediately after completing the disability awareness curriculum. For the MAS, the behaviors subscale failed to demonstrate a significant change within the medical student population, likely reinforcing that behavioral change is a process evolving from change in thought that requires additional time to manifest. After completion of all disability curricular elements, medical students’ overall and individual domain scores for both scales were similar to those of DPT students, which showed favorable attitudes prior to participating in CRWM and remained constant after the event. The lower baseline attitudes of medical students relative to their DPT counterparts are likely a function of the differing curricula of these health professions programs. Unlike US medical schools [31], DPT programs have multiple accreditation elements that specifically address disability [16] within the curriculum. Second-year DPT students who participated as interprofessional peer educators during CRWM had already completed most of the disability educational content in their professional degree program, and they had worked with PWD in both clinical and educational settings prior to this experience. Second-year medical students, however, had no formal curricular instruction around disability prior to these experiences. Qualitative analyses further supported improved medical student attitudes. Within the education theme, data indicated the students’ desires for continued learning about the care and medical management of PWD as well as ways to improve their access to medical care. Medical students further expressed that they would advocate for PWD and provide individualized care for them. In a recent study involving focus groups of PWD, participants emphasized the need for greater advocacy and political engagement to improve healthcare for PWD [32].

The use of proven educational strategies was essential to the positive outcomes of the disability awareness curriculum. Many components of the curriculum directly involved PWD as facilitators or presenters. For example, wheelchair users co-led each student group during CRWM, patients with specific disabilities interacted with students during the patient presentations, and some facilitators who led the Disability Debrief sessions had disabilities themselves. Direct interactions with PWD have been shown to provide an ideal learning environment for healthcare students [17], allowing students to become aware and better understand patients’ own expertise in living with their disabilities [33]. Intergroup contact theory (ICT) provides a framework to guide interactions between PWD and healthcare students [18, 33]. ICT states that intergroup contact under specific conditions can improve attitudes and reduce prejudice between majority and minority groups. Prior work has demonstrated the validity of ICT in augmenting healthcare students’ attitudes toward PWD [15, 34]. While disability simulations tend to distort what disability looks like by reinforcing outdated, ableist ideas, learning from PWD eliminates any falsely created representation of disability and allows for students to see what disability looks like as a lived experience [35].

Another educational strategy employed by this curriculum is peer-assisted learning (PAL). Medical students appear to benefit from instruction from those who are equivalent in terms of professional development [36, 37]. A systematic review demonstrated that peer-to-peer (students of the same cohort) and near-peer (students in the same profession but the more senior student teaches the junior student) instructions are effective [38]. Recent literature provides several successful examples of interprofessional PAL employed as a teaching strategy among healthcare students [20, 21]. For example, one study used DPT and occupational therapy students to effectively teach medical students about healthcare worker roles and responsibilities [39]. The current study further supports the implementation of interprofessional PAL. Coordinated utilization of PWD and interprofessional peers is worthy of further exploration.

Chardavoyne et al. [30] and Kirshblum and colleagues [34] have reported greater comfort among medical students when interacting with PWD after having received a disability curriculum in medical school. Results of this study, however, indicate medical students’ comfort may vary depending upon the type of disability. Medical students felt comfortable communicating with PWD in the domain of mobility but may have some discomfort in communicating with PWD who have communication deficits (theme, comfort; subtheme, negative). A recent research study also found that physicians working with more complex PWD who have communication deficits felt less comfortable especially when taking a history, performing a physical exam, and ascertaining a differential diagnosis [30]. Another study found that physicians have more difficulty caring for patients whose disability involves a communication impairment, including those with IDD [40]. Intentional training on how to communicate with individuals who have communication deficits would likely be helpful for medical students since IDD and hearing impairments comprise a significant portion of overall disability [2].

Medical students expressed concern regarding the lack of inclusion of specific diagnoses that are associated with developmental and physical disabilities, such as cerebral palsy, in their didactic curriculum. As medical schools continue to shorten the preclinical curriculum and reduce the breadth of specific diagnoses taught in the first 2 years, this concern may persist [41–43]. One unintended outcome of this disability awareness curriculum, though, was that many students engaged in self-directed learning to understand participant diagnoses of which they were unfamiliar. Self-directed learning is a required curricular element for allopathic medical schools [31] and is a core skill expected of graduating medical students. While a reduction in the number of specific diagnoses taught in shortened preclinical curricula may continue in UME, it will be important to ensure that experiential learning activities such as disability education promote transferable skills such as self-directed learning, empathy, and communication.

The evaluation of long-term outcomes of disability awareness curricula in medical education will be required to determine if improved comfort when interacting with PWD extends to medically caring for this population. A recent study showed that medical students who met individually with a PWD to complete a brief social and medical history were more confident, skilled, comfortable, efficient, and calm following the patient encounter [44]. Including experiences in which medical students perform medical care for PWD through simulated or real experiences may lead to better healthcare outcomes for this population.

This research study has several limitations. First, this curriculum was implemented at one medical school; therefore, results may not be generalizable to other medical students or institutions. Due to medical school curricular time constraints, intentional efforts were necessary to provide time for students to engage in these learning activities. While no other curricular elements were eliminated, the addition of these disability-focused sessions added additional mandatory contact time to the Musculoskeletal and Skin course. Second, the tools used to evaluate the curriculum are subject to self-report biases. Intentional data triangulation was conducted to minimize this potential and increase confidence in the findings reported. Third, the pre-test/post-test study design does not control for history, maturation, and testing biases. In this study, the risk of history and maturation biases are small due to a short timeframe between the intervention and testing. However, the questions in the surveys at pre-testing may have influenced medical student attitudes when answering questions at post-test. Future studies should use a randomized controlled trial design to eliminate these biases. Fourth, student participants in the focus groups were volunteers. As such, there is a risk of self-selection bias and focus group members may not necessarily be representative of the general population of medical students. And fifth, this study does not ascertain that the changes in attitudes by medical students immediately following the disability awareness curriculum are sustainable or carry into graduate medical education (GME) and clinical practice. This study does not measure the longitudinal aspect of this disability intervention and how these attitudes are translated following the post evaluation. Future studies should examine the sustainability of these attitudes over time.

Based on the results of this study and the authors’ experiences with implementing a disability awareness curriculum in UME, the following recommendations are provided for other medical schools seeking to develop a similar curriculum. First, the importance of involving PWD into the planning and implementation cannot be understated. Embedding the voice of PWD into the curriculum is critical to prevent the propagation of ableism. Second, disability is not a single entity; thus, diverse representation of PWD and disability type is important to ensure students appreciate the breadth of lived experiences. Faculty facilitators with clinical backgrounds that include working with PWD (e.g., physiatrists, physical therapists, occupational therapists) are important to ensure that important clinical considerations are stressed for students. Providing a facilitator guide is helpful to ensure each student group receives a similar experience. Finally, it is important to ensure learners have longitudinal opportunities to interact with patients who have disabilities so that they can practice the skills learned. Only through these real encounters will behavioral change truly be obtained.

## Conclusions

In conclusion, preliminary evidence suggests that our disability awareness curriculum improved medical students’ attitudes toward PWD. Qualitative evaluation has informed future improvements to the curriculum, such as dedicated training on communication skills for individuals with communication deficits. Further evaluation of this curriculum or similar disability curricula are needed to help decrease the gap of care between PWD and those without disability. This curriculum provides a framework for other medical schools seeking to implement a disability curriculum into UME.

### Supplementary Information

Below is the link to the electronic supplementary material.

Supplementary file1 (DOCX 17 KB)

## Data Availability

The datasets generated during and/or analyzed during the current study are available from the corresponding author on reasonable request.

## References

[1] Okoro CA (2018). Prevalence of disabilities and health care access by disability status and type among adults - United States, 2016. MMWR Morb Mortal Wkly Rep.

[2] CDC. Centers for Disease Control and Prevention. Disability and Health Data System (DHDS). 2023. https://www.cdc.gov/ncbddd/disabilityandhealth/infographic-disability-impacts-all.html. Accessed 01 Aug 2023.

[3] Long-Bellil LM (2011). Teaching medical students about disability: the use of standardized patients. Acad Med.

[4] Iezzoni LI (2021). Physicians’ perceptions of people with disability and their health care. Health Aff (Millwood).

[5] McMillan C (2016). Physician perspectives on care of individuals with severe mobility impairments in primary care in Southwestern Ontario. Canada Health Sco Care Community.

[6] Iezzoni LI (2021). Use of accessible weight scales and examination tables/chairs for patients with significant mobility limitations by physicians nationwide. Jt Comm J Qual Patient Saf.

[7] Lagu T (2022). ‘I am not the doctor for you’: Physicians’ attitudes about caring for people with disabilities. Health Aff (Millwood).

[8] Dixon-Ibarra A, Horner-Johnson W (2014). Disability status as an antecedent to chronic conditions: National Health Interview Survey, 2006–2012. Prev Chronic Dis.

[9] Friedman C (2023). Explicit and implicit: ableism of disability professionals. Disabil Health J.

[10] Seidel E, Crowe S (2017). The state of disability awareness in American medical schools. Am J Phys Med Rehabil.

[11] Holder M, Waldman HB, Hood H (2009). Preparing health professionals to provide care to individuals with disabilities. Int J Oral Sci.

[12] Englander R (2016). Toward defining the foundation of the MD degree: core entrustable professional activities for entering residency. Acad Med.

[13] Santoro JD (2017). Disability in US medical education: disparities, programmes and future directions. Health Educ J.

[14] Iezzoni LI (2006). Going beyond disease to address disability. N Engl J Med.

[15] Parish A (2022). Come roll with me: an interprofessional experience to promote disability awareness. Teach Learn Med..

[16] CAPTE. Commission on Accreditation in Physical Therapy Education. PT Standards and required elements. 2020. https://www.capteonline.org/globalassets/capte-docs/capte-pt-standards-required-elements.pdf. Accessed 01 Aug 2023.

[17] Shakespeare T, Iezzoni LI, Groce NE (2009). Disability and the training of health professionals. Lancet.

[18] Pettigrew TF, Tropp LR (2006). A meta-analytic test of intergroup contact theory. J Pers Soc Psychol.

[19] Sadowski CA (2015). Interprofessional peer teaching of pharmacy and physical therapy students. Am J Pharm Educ.

[20] Hsia S (2020). Interprofessional peer teaching: the value of a pharmacy student-led pharmacology course for physical therapy students. Curr Pharm Teach Learn.

[21] Dunleavy K, Sposetti V. Interprofessional peer teaching: physical therapy students teaching dental students to transfer patients. Collaborative Healthcare: Interprofessional Practice, Education and Evaluation. 2017;7(2).

[22] Brooks WS (2015). Integration of gross anatomy in an organ system-based medical curriculum: strategies and challenges. Anat Sci Educ.

[23] Savarirayan R (2020). Once-daily, subcutaneous vosoritide therapy in children with achondroplasia: a randomised, double-blind, phase 3, placebo-controlled, multicentre trial. Lancet.

[24] Savarirayan R (2021). Safe and persistent growth-promoting effects of vosoritide in children with achondroplasia: 2-year results from an open-label, phase 3 extension study. Genet Med.

[25] Myong Y (2021). Development and validation of a new scale to assess attitudes and perspectives toward persons with disabilities. Ann Rehabil Med.

[26] Findler L, Vilchinsky N, Werner S (2007). The multidimensional attitudes scale toward persons with disabilities (MAS): construction and validation. RCB.

[27] Mills J, Bonner A, Francis K (2006). The development of constructivist grounded theory. Int J Qual Methods.

[28] Denzin NK, Lincoln YS. The landscape of qualitative research. 3rd ed. Newbury Park, CA: Sage Publications, Inc.; 2008.

[29] Brooks WS (2024). Survey response rates in health sciences education research: a 10-year meta-analysis. Anat Sci Educ.

[30] Chardavoyne PC, Henry AM, Sprow Forte K (2022). Understanding medical students’ attitudes towards and experiences with persons with disabilities and disability education. Disabil Health J.

[31] LCME. Liaison Committee on Medical Education. Functions and structure of a medical school. 2023. https://lcme.org/publications/. Accessed 01 Aug 2023.

[32] McClintock HF (2018). Health care access and quality for persons with disability: patient and provider recommendations. Disabil Health J.

[33] Pettigrew TF (1998). Intergroup contact theory. Annu Rev Psychol.

[34] Kirshblum S (2020). An introductory educational session improves medical student knowledge and comfort levels in caring for patients with physical disabilities. Disabil Health J.

[35] Nario-Redmond MR, Gospodinov D, Cobb A (2017). Crip for a day: the unintended negative consequences of disability simulations. Rehabil Psychol.

[36] Khaw C, Raw L (2016). The outcomes and acceptability of near-peer teaching among medical students in clinical skills. Int J Med Educ.

[37] Nelson AJ (2013). Tomorrow’s educators … today? Implementing near-peer teaching for medical students. Med Teach.

[38] Burgess A, McGregor D, Mellis C (2014). Medical students as peer tutors: a systematic review. BMC Med Educ.

[39] Dunleavy K (2017). Impact of interprofessional peer teaching on physical and occupational therapy student’s professional role identity. J Interprofessional Educ Pract.

[40] Bachman SA (2006). Provider perceptions of their capacity to offer accessible health care for people with disabilities. J Disabil Policy Studies.

[41] McDaniel CM, Forlenza EM, Kessler MW (2020). Effect of shortened preclinical curriculum on medical student musculoskeletal knowledge and confidence: an institutional survey. J Surg Educ.

[42] Held N (2023). Designing a shortened preclinical basic science curriculum: expert-derived recommendations. Acad Med.

[43] Elfanagely Y (2020). How curricular changes influence medical students’ perceptions of basic science: A pilot study. PLoS ONE.

[44] Crane JM (2021). Getting comfortable with disability: the short- and long-term effects of a clinical encounter. Disabil Health J.

