# Supplementary material for: Integration of Disability Awareness Improves Medical Students’ Attitudes Toward People with Disabilities
Source: Med Sci Educ. 2024 Mar 9;34(3):561–9. doi: 10.1007/s40670-024-02004-0 (PMC11180063; doi:10.1007/s40670-024-02004-0)
Supplement: Supplementary file 1 — Supplementary file1 (DOCX 17 KB) [file 40670_2024_2004_MOESM1_ESM.docx]

**Project Title:** Interprofessional Experiences Between Medical and Physical Therapy Students and Individuals with Disabilities

**Focus Session**: Medical Student Perspectives on Disability Awareness Training

**Part A. Inclusion Screening**

You were selected to participate in today’s focus session because you participated in a variety of disability awareness activities during the 2022 Musculoskeletal & Skin Module in the Heersink School of Medicine.

**Part B: “Ground Rules” for Focus Groups**

My colleagues and I are interested in your perceptions of the disability education activities that were part of the Musculoskeletal & Skin Module, including Come Roll with Me, United Ability, Disability Training, and Disability Debrief. This information will be used to help us make this course better next year but is also being collected for research purposes. If this data is published in a manuscript, we will not use any participant information to protect your identity. We believe that these teaching strategies may be an effective method to promote disability awareness to other professionals and other medical students in other schools. Your input will help us determine if this belief is true or not.

If you are a UAB employee, please know that your employment status will not be affected by participation in this study. Similarly, your status and opportunities as a student will not be affected by participation. Participation is voluntary.

There are no right or wrong answers, and you are not obligated to answer any questions should you choose. Your participation and responses will remain confidential, you can withdraw at any time without penalty, and your status and opportunities as a UAB student will not be affected by your participation. The group discussion should take no more than one hour.

**Part B. Semi-Structured Focus Group Questions**

1. What elements of Come Roll with Me were most impactful to you?

Possible probe questions:

- As a reminder, you learned how to perform transfers, you learned about vehicles and parking, you had a conversation with a wheelchair user about their daily life, and you self-propelled yourself in a wheelchair.
- Why was this element impactful?
- Can you be more specific?
- Can you tell me more about that?

2. What elements of your experience at United Ability were most impactful to you?

Possible probe questions:

- Why was this element impactful?
- Can you be more specific?
- Can you tell me more about that?

3. What other curricular activities within the MSK-Skin Module affected your perceptions of individuals with disabilities?

Possible probe questions:

- As a reminder, you received a lecture on disability, you participated in a disability debrief small group session with your peers, and you attended achondroplasia and myopathies patient presentations.
- Why was this element impactful?
- Can you be more specific?
- Can you tell me more about that?

4. Of all the various activities in the MSK-Skin module that you participated in, which one do you feel had the greatest impact on influencing how you perceive people with disabilities?

Possible probe questions:

- Can you be more specific?
- Can you tell me more about that?

5. Did any of the activities in the MSK-Skin module have a negative impact on how you perceive people with disabilities?

Possible probe questions:

- Can you be more specific?
- Can you tell me more about that?

6. How did your interactions with physical therapy students and faculty during Come Roll with Me and Simulation impact you?

Possible probe questions:

- Can you be more specific?
- Can you tell me more about that?

7. What aspects of disability did you learn the most about during the MSK-Skin Module?

Possible probe questions:

- Can you be more specific?
- Can you tell me more about that?

8. What aspects of disability do you wish had been included in the course but were not?

Possible probe questions:

- Can you be more specific?
- Can you tell me more about that?

9. What aspects of the course and the disability awareness events do you feel could be changed or improved for the future?

Possible probe questions:

- Can you be more specific?
- Can you tell me more about that?
- What change of the ones you have proposed would be the most important to implement?

*Thank you so much for your time and support of this project.*
